# Comparison of AI‐based retinal fluid monitoring in neovascular age‐related macular degeneration with manual assessment by different eye care professionals under optimized conditions

**DOI:** 10.1111/aos.17458

**Published:** 2025-02-14

**Authors:** Martin Michl, Bianca S. Gerendas, Anastasiia Gruber, Felix Goldbach, Georgios Mylonas, Oliver Leingang, Wolf Bühl, Stefan Sacu, Hrvoje Bogunovic, Amir Sadeghipour, Ursula Schmidt‐Erfurth

**Affiliations:** ^1^ Department of Ophthalmology and Optometry Medical University Vienna Vienna Austria; ^2^ Center for Medical Statistics Medical University Vienna Vienna Austria; ^3^ RetInSight GmbH Vienna Austria

**Keywords:** automated, grading, manual, neovascular age‐related macular degeneration, optical coherence tomography, retinal fluid

## Abstract

**Purpose:**

To investigate whether automated intra‐ and subretinal fluid (IRF/SRF) volume measurements are equivalent to manual evaluations by eye care professionals from different backgrounds on real‐world optical coherence tomography (OCT) images in neovascular age‐related macular degeneration (nAMD).

**Methods:**

Routine OCT images (Spectralis, Heidelberg Engineering) were obtained during standard‐of‐care anti‐VEGF treatment for nAMD at a tertiary referral centre. IRF/SRF presence and change (increase/decrease/stability) were assessed without time constraints by five retinologists, three ophthalmology residents, three general ophthalmologists, three orthoptists and three certified readers. Fluid volumes were segmented and quantified using a regulatory‐approved AI‐based tool (Vienna Fluid Monitor, RetInSight, Vienna, Austria). Sensitivity/specificity (Sen/Spe) for grading fluid presence and kappa agreement were calculated for each group. Their performances in distinguishing between IRF/SRF increase and decrease were assessed using AUCs.

**Results:**

About 124 follow‐up visit pairs of 59 eyes with active nAMD were included. Across all five groups, fluid volumes >5 nL were identified with values of 0.81–0.95 (Sen)/0.70–0.91 (Spe) for IRF and 0.89–0.98 (Sen)/0.74–0.90 (Spe) for SRF. Interpretations of IRF changes between −17 nL and +3 nL and SRF changes between −9.30 nL and +6.50 nL were associated with Sen > 0.80 and Spe > 0.87 among all groups. Agreements between the algorithm and groups in grading IRF/SRF presence ranged from *κ* = 0.69–0.82/0.73–0.79. The AUC for correctly classifying fluid change was >0.89 across all groups.

**Conclusion:**

Eye care professionals with different levels of clinical expertise assessed disease activity on standard OCT images with comparable accuracy. Despite optimizing the methodology and time resources, manual performance did not reach the high level of automated fluid monitoring.

## 1 INTRODUCTION

The current human expert assessment of disease activity in exudative retinal diseases such as neovascular age‐related macular degeneration (nAMD) is not only subjective and time‐intensive but also focuses primarily on the presence/absence of macular fluid, as seen on optical coherence tomography (OCT) images. Although routine assessment is performed by a wide spectrum of professionals on a daily basis, there is evidence of substantial grading disagreement among clinicians that might negatively impact treatment decisions and consecutively patients' vision (DeCroos et al., 2012; Muller et al., 2021; Toth et al., 2015). This is particularly critical, as there is an increasing demand for anti‐vascular endothelial growth factor (VEGF) injections in the treatment of nAMD due to the growing proportion of elderly individuals (Wong et al., 2014). There is thus an urgent need to facilitate access to diagnostic tests that are easy to perform and produce robust and clinically relevant information in a transparent and comprehensible way.

In real‐life clinical practice, the treat‐and‐extend (T&E) regimen has become the mainstay for the management of nAMD patients and is largely based on the presence of intra‐ (IRF) and subretinal fluid (SRF). In contrast to pro‐re‐nata (PRN) regimens, T&E was introduced as a proactive, individualized approach that prevents any appearance of recurrent fluid. By adjusting treatment intervals between injections in the absence of disease recurrence, the strategy aims at reducing the monitoring burden on patients and caregivers to a minimum (Lanzetta & Loewenstein, 2017; Spooner et al., 2018). However, to optimally tailor treatment intervals to patients' needs and avoid under‐ and overtreatment, accurate personalized management should take into account the dynamics and functional implications of retinal fluid in the individual patient (Chakravarthy et al., 2021; Guymer et al., 2019; Riedl et al., 2022). This requires a precise and objective identification of retinal fluid levels and their changes over the course of the disease by the responsible caretaker. There is yet no assessment available that evaluates the manual grading capabilities of personnel who are routinely involved in identifying and treating active nAMD. Furthermore, previous studies have shown a clear association between retinal fluid levels and fluctuations with visual function, but whether such changes are actually distinguishable by human experts has not been established (Chakravarthy et al., 2021; Reiter et al., 2021, 2024; Riedl et al., 2022).

Artificial intelligence (AI)‐based fluid quantification is a promising tool that allows an accurate and time‐efficient assessment while minimizing subjectivity and bias (Aziz et al., 2024; Coulibaly et al., 2022). The aim of this study was to compare the accuracy of eye care professionals in assessing IRF/SRF presence and change under ideal conditions regarding methodology and time resources with automatically measured volumetrics using an AI‐based tool from standard‐of‐care in nAMD.

## 2 METHODS

### 2.1 Population/Dataset

OCT data as well as demographic and treatment information were retrieved from medical records of more than 25 000 patients who were treated at the outpatient retina clinic of the Medical University of Vienna between 2007 and 2018 and are part of the VIBES registry (Vienna Imaging Biomarker Eye Study, Medical University of Vienna, Austria). The anti‐VEGF agents used were ranibizumab and aflibercept according to the approved label indications. We included OCT scans of nAMD patients who received Spectralis OCT (Heidelberg Engineering, Heidelberg, Germany) imaging at two consecutive visits. A 6 × 6 mm macular cube scan pattern with a resolution of 512 × 49 was used. The allowed time between the two visits was 28–120 days, during which the patient had to receive an anti‐VEGF injection. Excluded were scans if both of the following two criteria were met: (1) the IRF/SRF volume was <5 nL at visit 1 and (2) the IRF/SRF volume change to visit 2 was <5 nL, as quantified by the automated fluid algorithm. The Ethics Committee of the Medical University of Vienna approved this registry and analysis (approval numbers: 2094/2018 & 2095/2018). The study adhered to the tenets of the Declaration of Helsinki and the principles of Good Scientific Practice.

### 2.2 Automated IRF/SRF segmentation

**FIGURE 1:**
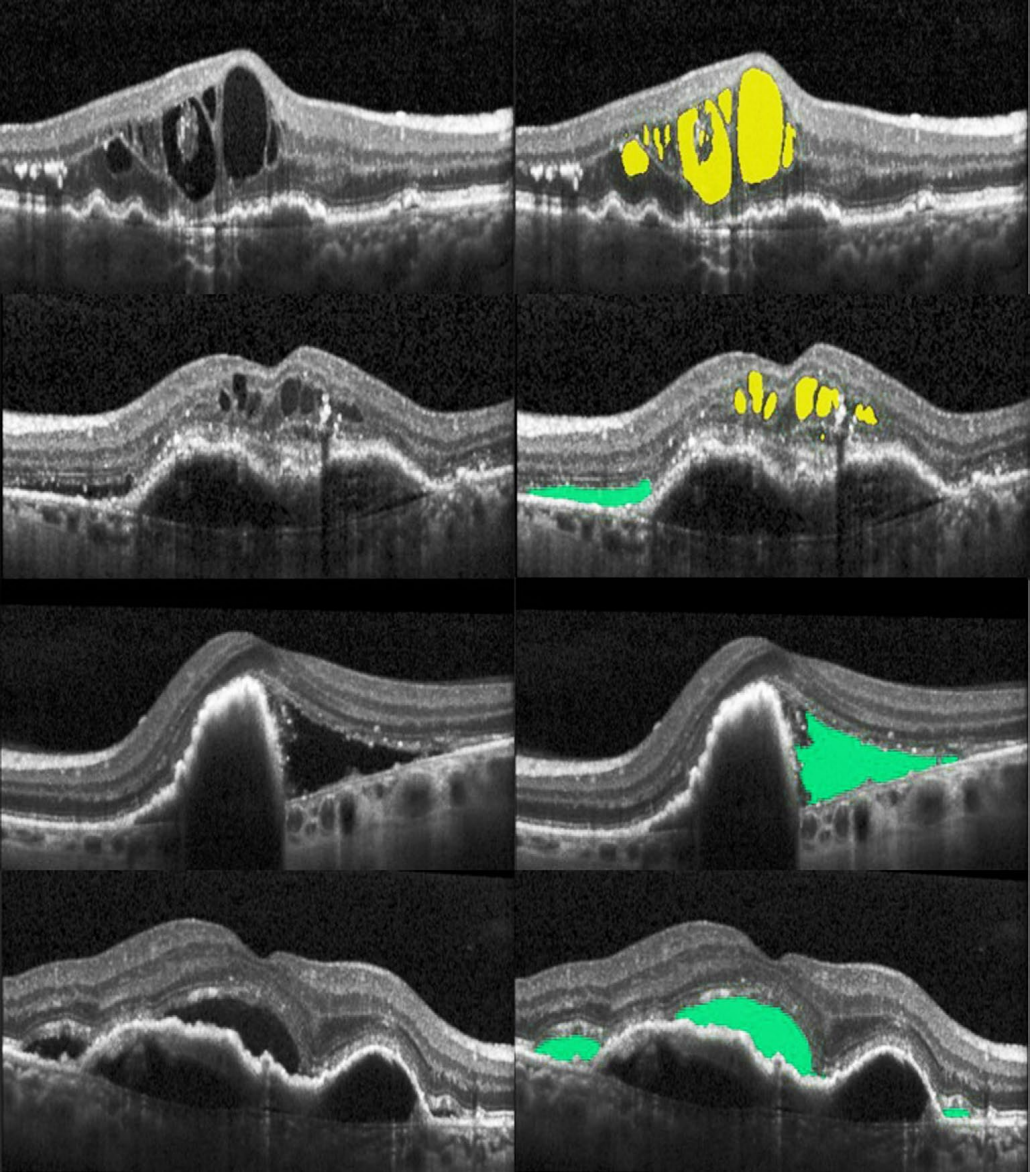
Representative examples of spectral‐domain OCT scans from nAMD patients. B‐scans in the left column are shown without fluid segmentations, in the right with segmentations from the deep learning fluid monitor. Intraretinal fluid is shown in yellow, subretinal fluid in green.

OCT images were segmented using the Vienna Fluid Monitor (V1.3, RetInSight, Austria), a deep‐learning‐based, medical device regulation (MDR) (European Union)‐approved algorithm (2017/745) for the automated detection of retinal fluid in nAMD. Using a convolutional neural network, the algorithm automatically segments three‐dimensional OCT volumes and predicts the likelihood of belonging to one of the four classes (IRF, SRF, retina, or background) for every pixel. For the training of the algorithm, expert graders had performed pixelwise fluid annotation under the supervision of retina specialists (Schlegl et al., 2018). The model has been extensively tested and validated on various large datasets, including trials and real‐world patient cohorts (Gerendas et al., 2022; Grechenig et al., 2021; Mares et al., 2024; Reiter et al., 2021; Schmidt‐Erfurth et al., 2023). As an additional quality measure in this study, OCT image quality and the overall correctness of automated IRF/SRF segmentations in the total scan area were manually controlled in all scans by two ophthalmologists (MM, FG) from the Vienna Reading Centre with experience in OCT grading and supervision. Figure 1 shows representative B‐scans with segmented fluid compartments.

### 2.3 Manual grading of OCT scans

A total of 17 eye care professionals participated as human expert graders in this study: five retinologists (RET), three ophthalmology residents (RES), three general ophthalmologists working in primary eye care outside of a hospital setting (GENO), three orthoptists (ORTH) and three graders from the Vienna Reading Centre (VRC). In a custom software, each grader first centred the OCT scan to the fovea, thereby aligning scan positions at both visits and allowing simultaneous side‐by‐side presentation. Graders were then able to navigate through the scans and assess the presence of IRF/SRF in the entire OCT volume scan at both consecutive visits. In case IRF/SRF was identified by the grader on either scan, its change to the subsequent visit (increase/decrease/no change – stable) was documented. Segmentations by the automated fluid monitor were not shown in the masked manual grading process. The graders were masked to all patient information and each other's assessments. They received no specific OCT training prior to the study and there was no time limit to complete the tasks.

### 2.4 Statistical analysis

Automatically measured fluid volume distributions are presented as medians and interquartile ranges (IQRs). For the calculation of sensitivity (Sen) and specificity (Spe) in grading *IRF/SRF presence* at each visit, the majority vote of each of the five professional groups was used. Automatically measured fluid volumes were then used to quantitatively describe each professional group's decision on fluid presence for every visit. Fluid presence according to the fluid monitor was defined as a volume >5 nL in the total scan area, and Sen/Spe were calculated for each professional group.

To determine the classification performance in distinguishing *IRF/SRF changes* (increase vs. decrease) in a given visit pair, receiver operating characteristic (ROC) curves were generated. The optimal volume threshold with maximum values for Sen/Spe was defined by the Youden index. Area under the ROC curve (AUC) values were calculated as measures of overall *classification performance*.

To identify potential biases and illustrate the robustness of the fluid monitor, the *grading agreement* on IRF/SRF presence between the individual groups and the fluid monitor was assessed using Fleiss' kappa and its 95% confidence intervals (CIs).

The software ‘R' was used for all calculations of Sen/Spe as well as for the generation of ROCs and Fleiss' Kappa values.

## 3 RESULTS

A total of 124 visit pairs of 59 eyes from 57 individuals were included (left eyes: 28; 47.5%). The mean patient age was 74.5 years (SD: 10.4). There were 39 women (68.4%) and 18 men (31.6%) in our study cohort.

One visit pair had to be excluded due to different image sizes of the two OCT scans, making a reliable evaluation by the graders impossible. No scan had to be excluded because of erroneous fluid segmentations.

### 3.1 Disease activity characteristics

**FIGURE 2:**
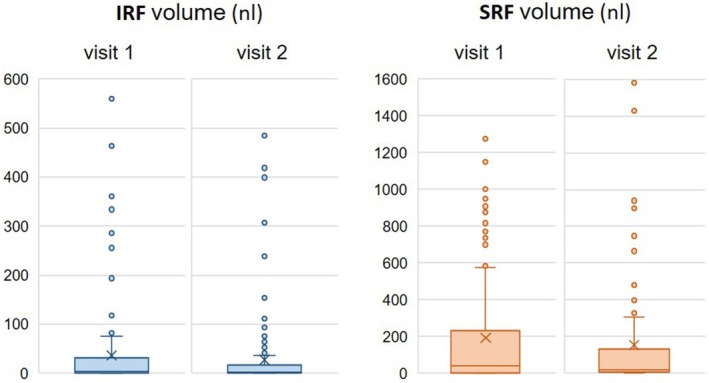
Distribution of intra‐ (IRF) and subretinal fluid (SRF) volumes (nL) at visits 1 and 2. ‘*X*' represents the mean of the data. Outliers are shown as dots outside the whiskers.

Based on automated volume assessment, the median IRF volumes were 3.62 nL (IQR: 0.52–33.31) at visit 1 and 1.82 nL (0.32–16.86) at visit 2 (the small median IRF volumes are due to visits that were included based on their sufficiently large SRF volumes, while containing <5 nL IRF = minimum volume to avoid exclusion). The measured median SRF volume was 45.29 nL (3.01–260.75) at visit 1 and 12.14 nL (0.82–125.55) at visit 2 (Figure 2). The median change between two consecutive visits was 0 nL (−1.36 to +8.88) for IRF and 6.18 nL (−7 to +73.3) for SRF.

### 3.2 Comparison of human with automated grading

Comparing the volumetrics to the human grading allowed a quantitative description of the individual groups' decisions on fluid presence and change (Table 1). Notably, the median volume in patients for whom each group's vote was ‘IRF/SRF not present’ was <0.70 nL/0.40 nL. The median fluid change was <1 nL in cases where the vote was ‘no change/stable fluid’. For IRF/SRF change, the number of visit pairs where no majority vote was reached within a group was 1/2 for RET, 1/0 for RES, 3/1 for GENO, 3/1 for ORTH, and 6/0 for VRC. There were no disagreements on fluid presence/absence (dichotomous variable).

**TABLE 1:** Automated fluid quantifications (nL) of the individual groups' decisions on fluid presence and change.

a.	IRF presence				SRF presence			
	‘Yes’	*n*	‘No’	*n*	‘Yes’	*n*	‘No’	*n*
RET	25.45 (10.80 to 69.02)	114	0.59 (0.03 to 1.63)	134	68.90 (13.28 to 312.26)	175	0.07 (0 to 1.61)	73
RES	28.32 (11.26 to 69.02)	117	0.59 (0.03 to 1.61)	131	93.44 (18.25 to 335.76)	165	0.18 (0 to 1.97)	83
GENO	26.87 (10.00 to 68.78)	119	0.61 (0.03 to 1.81)	129	107.37 (27.38 to 391.02)	157	0.40 (0 to 2.41)	91
ORTH	31.78 (14.19 to 75.83)	109	0.66 (0.04 to 1.88)	139	102.10 (27.88 to 378.64)	160	0.29 (0 to 2.10)	88
VRC	19.55 (4.78 to 57.14)	116	0.48 (0.00 to 1.13)	132	68.25 (13.61 to 311.31)	183	0.05 (0 to 0.72)	65

b.	IRF change							
	‘Increase’	*n*	‘Decrease’	*n*	‘No change’	*n*	No agreement	*n*
RET	35.30 (8.73 to 198.58)	18	−53.96 (−156.80 to −29.20)	27	0 (−0.92 to +0.39)	78	−15.41 (−15.41 to −15.41)	1
RES	15.77 (7.44 to 129.90)	22	−35.05 (−114.83 to −8.88)	38	0 (−0.38 to +0.36)	63	−1.99 (−1.99 to −1.99)	1
GENO	20.68 (7.32 to 151.32)	21	−48.49 (−119.22 to −19.16)	31	0 (−0.64 to +0.44)	69	−5.49 (−17.36 to −2.60)	3
ORTH	35.30 (5.46 to 167.07)	16	−48.89 (−119.56 to −18.76)	30	0 (−0.67 to +0.47)	75	−12.29 (−33.10 to +209.77)	3
VRC	35.30 (7.66 to 198.58)	18	−35.05 (−117.57 to −9.05)	36	0 (−0.46 to +0.30)	64	6.88 (2.12–14.40)	6
	**SRF change**							
RET	98.05 (13.06 to 395.91)	34	−88.55 (−419.78 to −28.40)	54	−0.07 (−3.42 to +0.49)	34	23.70 (−13.80 to +61.21)	2
RES	123.79 (11.96 to 418.97)	33	−75.72 (−418.14 to −27.47)	57	−0.01 (−0.81 to +1.62)	34	—	—
GENO	123.79 (16.38 to 326.73)	29	−75.72 (−418.14 to −26.72)	57	−0.06 (−3.92 to +0.76)	37	0.73 (0.73–0.73)	1
ORTH	148.16 (16.73 to 395.91)	30	−92.29 (−428.44 to −33.20)	50	−0.06 (−6.09 to +0.94)	43	−49.87 (−49.87 to −49.87)	1
VRC	66.85 (7.07 to 326.73)	37	−72.40 (−371.18 to −26.41)	60	−0.01 (−0.54 to +0.64)	27	—	—

*Note*: Median fluid volumes (nL) and their IQRs, as quantified by the fluid monitor, for each group's decision regarding (a.) fluid presence/absence in both visits and (b.) fluid change between visits. The column ‘*n*’ highlights the number of cases, where a majority vote was reached within a group; the column ‘no agreement’ shows fluid volumes of cases where no such vote was reached. There were five graders in the group ‘retinologists’ and three graders in each of the other four groups.

Abbreviations: GENO, general ophthalmologists; IRF, intraretinal fluid; *n*, number of cases where an agreement was reached within the group; ORTH, orthoptists; RES, residents; RET, retinologists; SRF, subretinal fluid; VRC, reading centre graders.

### 3.3 Statistical analysis of fluid volumes

*IRF/SRF presence*. When compared with the fluid monitor, Sen/Spe in detecting IRF/SRF were comparable between professional groups: IRF: RET = 0.91/0.87, RES = 0.95/0.88, GENO = 0.91/0.84, ORTH = 0.89/0.91, VRC = 0.81/0.76; SRF: RET = 0.95/0.78, RES = 0.94/0.87, GENO = 0.89/0.88, ORTH = 0.92/0.90, VRC = 0.98/0.74.

*IRF/SRF change*. According to the Youden index (the smallest volume detected with the highest Sen and highest Spe), IRF increases between 1.70 nL and 2.90 nL were graded with 0.91–1.0 (Sen) and 0.88–0.93 (Spe) across all groups. Among retinologists (RET), an IRF decrease of 17 nL was graded with 0.89 (Sen) and 0.96 (Spe); among the other groups, IRF decreases between −2.80 nL and −7.20 nL were graded with 0.80–0.90 (Sen) and 0.90–0.96 (Spe). Regarding SRF increases, RET and VRC graded increases <1 nL with 0.92–0.94 (Sen) and 0.89–0.91 (Spe); the other groups detected SRF increases of 6.50 nL with 0.85–0.90 (Sen) and 0.94–0.96 (Spe). Regarding SRF decreases, slightly larger volumes of −6.40 nL to −9.30 nL were graded with 0.93–0.98 (Sen) and 0.87–0.95 (Spe) among all groups.

**FIGURE 3:**
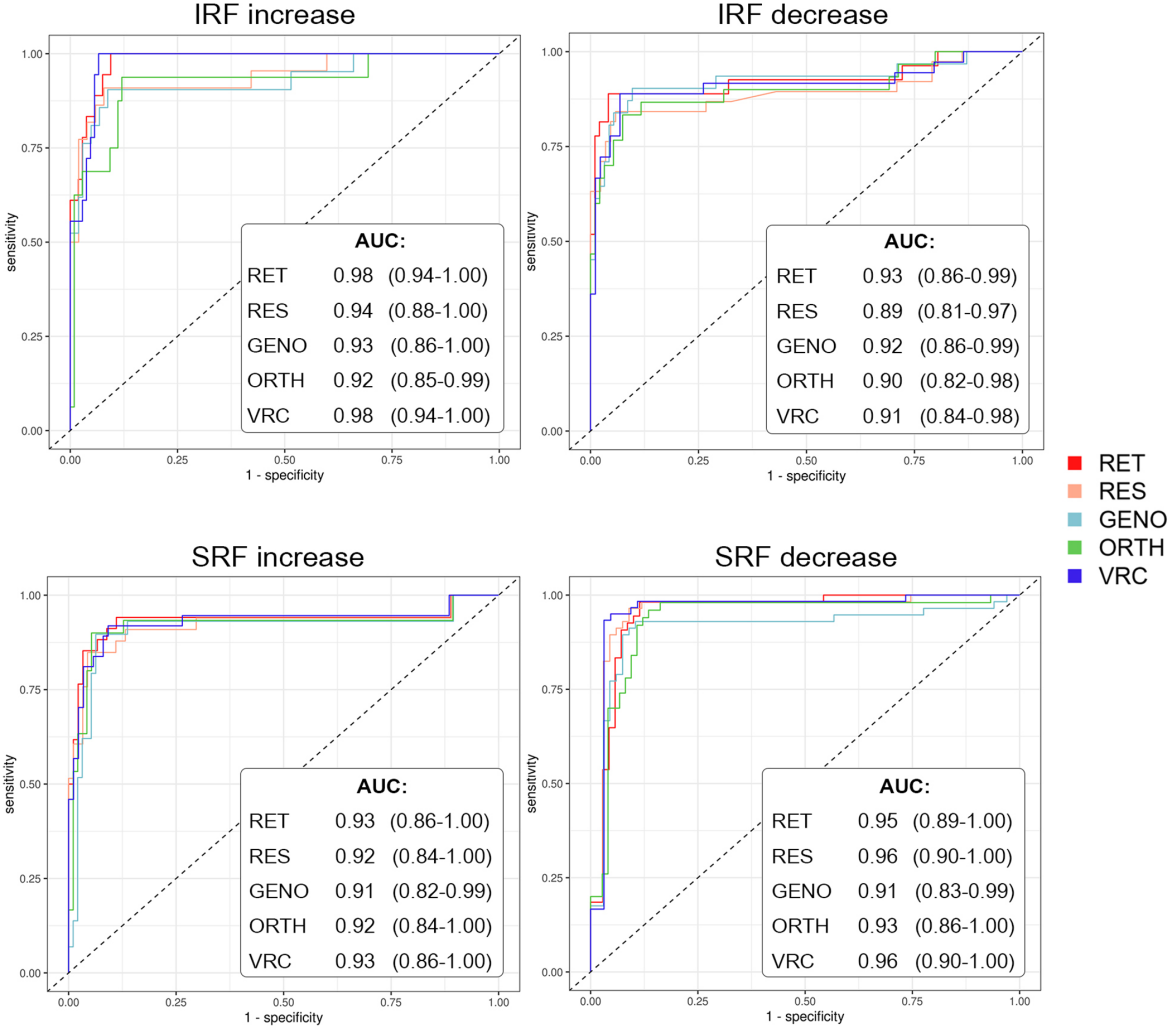
Classification performance for fluid change of the five professional groups. The receiver operating characteristic (ROC) curves show the classification performance of each professional group in discriminating between eyes with intra‐ (IRF) and subretinal fluid (SRF) increase and decrease. Dashed lines indicate chance performance. Each table in the bottom right corner lists the corresponding area under the curve (AUC) values and their confidence intervals.

**FIGURE 4:**
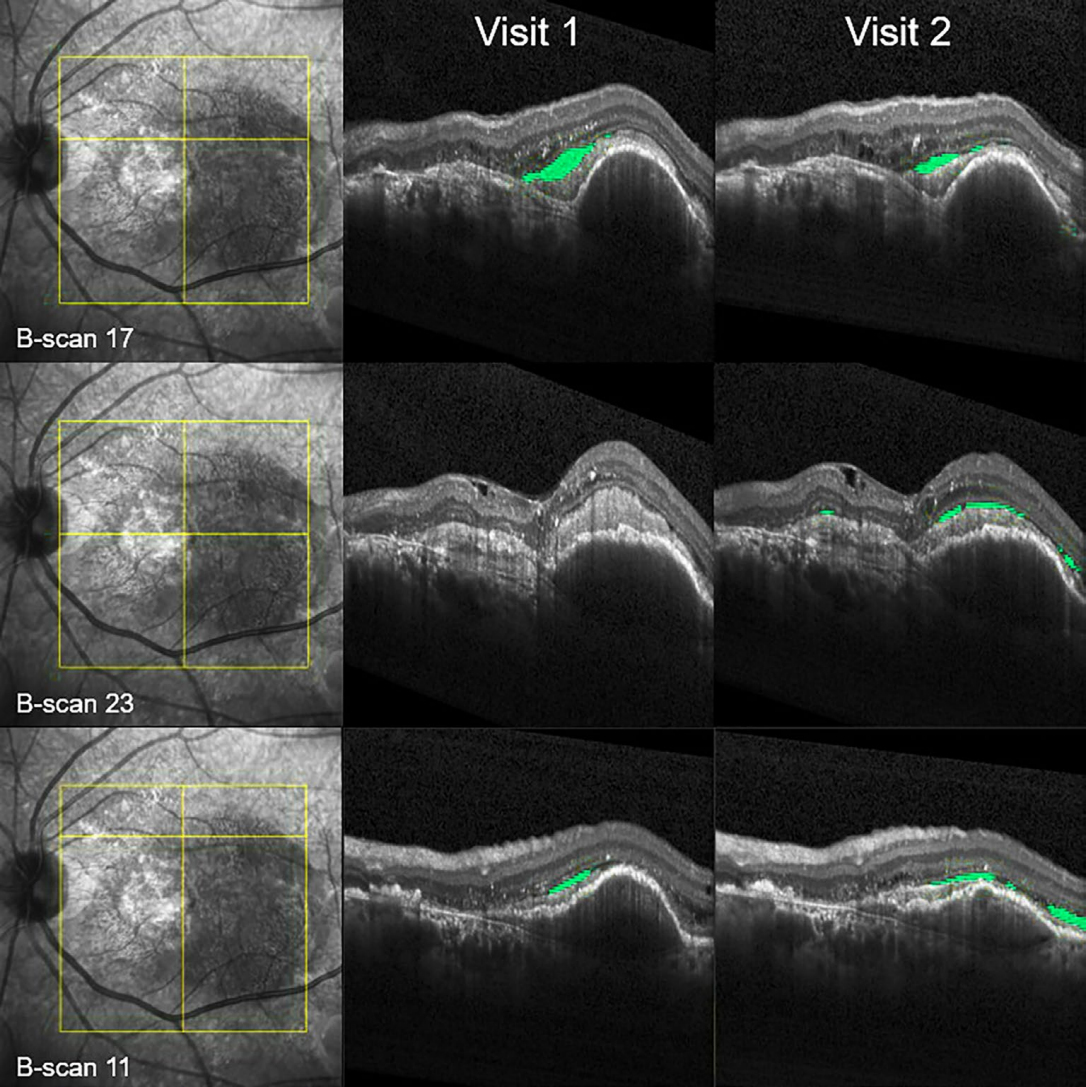
Example of a grading disagreement between the human graders and the fluid monitor. Spectral‐domain OCT B‐scans of a patient at three corresponding positions (three rows) at two consecutive visits (columns 2 and 3). Although there was a subretinal fluid (SRF) increase of approx. 100 nL from visit 1 to visit 2, as quantified by the fluid monitor (green segmentations), most graders indicated an SRF decrease. This discrepancy might be due to the obvious reduction of a larger SRF pocket seen in the first row as well as an underestimation of multiple narrow and elongated SRF pockets at visit 2, as shown in the exemplary images in rows 2 and 3.

The *classification performance* of each professional group in distinguishing between eyes with IRF/SRF increase or decrease was high, as indicated by an AUC >0.89 (Figure 3). Figure 4 depicts OCT images of a visit pair with a discrepancy between the groups' decisions and measurements from the fluid monitor.

### 3.4 Agreement between groups and fluid monitoring

As given in Table 2, the agreement for IRF presence/absence ranged from *κ* = 0.69 to 0.82 between the fluid monitor and all professional groups and from *κ* = 0.70 to 0.87 between the individual groups. For SRF presence/absence, the agreement ranged from *κ* = 0.73 to 0.79 between the fluid monitor and all groups and between *κ* = 0.69 to 0.89 between the professional groups only (Table 2). The grading agreements between graders within a professional group is shown in italics in Table 2.

**TABLE 2:** Kappa agreements for fluid presence/absence between the professional groups and the fluid monitor.

	FM	ORTH	GENO	VRC	RES	RET
**IRF presence**						
FM	—	0.81 (0.73–0.89)	0.74 (0.65–0.83)	0.69 (0.60–0.78)	0.82 (0.75–0.89)	0.78 (0.70–0.87)
ORTH	0.81 (0.73–0.89)	*0.73 (0.66–0.81)*	0.84 (0.77–0.91)	0.70 (0.61–0.79)	0.85 (0.78–0.92)	0.83 (0.76–0.90)
GENO	0.74 (0.65–0.83)	0.84 (0.77–0.91)	*0.78 (0.71–0.86)*	0.79 (0.71–0.87)	0.83 (0.76–0.90)	0.87 (0.80–0.93)
VRC	0.69 (0.60–0.78)	0.70 (0.61–0.79)	0.79 (0.71–0.87)	*0.58 (0.50–0.65)*	0.78 (0.70–0.86)	0.76 (0.68–0.84)
RES	0.82 (0.75–0.89)	0.85 (0.78–0.92)	0.83 (0.76–0.90)	0.78 (0.70–0.86)	*0.86 (0.78–0.93)*	0.86 (0.79–0.92)
RET	0.78 (0.70–0.87)	0.83 (0.76–0.90)	0.87 (0.80–0.93)	0.76 (0.68–0.84)	0.86 (0.79–0.92)	*0.79 (0.75–0.83)*
**SRF presence**						
FM	—	0.79 (0.71–0.88)	0.78 (0.69–0.86)	0.73 (0.64–0.83)	0.77 (0.69–0.86)	0.74 (0.64–0.83)
ORTH	0.79 (0.71–0.88)	*0.56 (0.48–0.63)*	0.83 (0.76–0.91)	0.77 (0.69–0.86)	0.89 (0.82–0.95)	0.75 (0.66–0.84)
GENO	0.78 (0.69–0.86)	0.83 (0.76–0.91)	*0.61 (0.53–0.68)*	0.69 (0.60–0.79)	0.83 (0.75–0.90)	0.74 (0.64–0.83)
VRC	0.73 (0.64–0.83)	0.77 (0.69–0.86)	0.69 (0.60–0.79)	*0.67 (0.60–0.75)*	0.81 (0.72–0.89)	0.87 (0.80–0.94)
RES	0.77 (0.69–0.86)	0.89 (0.82–0.95)	0.83 (0.75–0.90)	0.81 (0.72–0.89)	*0.73 (0.65–0.81)*	0.81 (0.73–0.89)
RET	0.74 (0.64–0.83)	0.75 (0.66–0.84)	0.74 (0.64–0.83)	0.87 (0.80–0.94)	0.81 (0.73–0.89)	*0.69 (0.65–0.74)*

*Note*: Fleiss' kappa values and their 95% CIs as measures of agreement between the individual expert groups and the fluid monitor. The presence of fluid in the fluid monitor was defined as >5 nL. Numbers in italics indicate the agreement between graders within a professional group.

Abbreviations: FM, fluid monitor; GENO, general ophthalmologists; IRF, intraretinal fluid; ORTH, orthoptists; RES, residents; RET, retinologists; SRF, subretinal fluid; VRC, reading centre graders.

## 4 DISCUSSION

In this study, using OCT images from a standard‐of‐care clinical routine, we compared the grading of retinal fluid performed by a wide spectrum of eye care professionals under optimal conditions to that of a validated and MDR‐certified deep‐learning‐based fluid monitor. This is the first time such an automated tool has been used as a reference standard to quantitatively define the accuracy of human graders in assessing the presence and change of IRF/SRF in patients with exudative retinal disease. All 17 eye care professionals included in this study are routinely involved in the management of such diseases, from the acquisition of images and the identification of exudative activity (both in the clinical routine and clinical trials) to the clinical referral for intravitreal injections and/or the final treatment decision.

In terms of grading fluid presence (defined as >5 nL by the fluid monitor), we found that eye care professionals were able to complete this task with comparable accuracy, reaching Sen/Spe values >0.81/0.76 for the identification of IRF and 0.89/0.74 for SRF. Even between the most experienced graders, that is, retinologists, who evaluate OCT images on a daily basis, and orthoptists, who, at our clinic, are merely involved in the acquisition but not the interpretation of OCT data, there was no apparent difference in grading reliability.

While grading agreements for IRF/SRF presence among retina specialists and reading centre personnel have previously been described in the ranges of *κ* = 0.48–0.69 (IRF) and *κ* = 0.80–0.82 (SRF), this is the first time that orthoptists, residents and general ophthalmologists were included in such an analysis (DeCroos et al., 2012; Michl et al., 2023; Muller et al., 2021). The agreements of *κ* = 0.70–0.87 (IRF) and *κ* = 0.69–0.89 (SRF) across all groups as well as their comparable reliability in identifying retinal fluid suggest that clinical experience might only play a limited role in assessing defined morphological changes on B‐scans of standard OCT volumes. Importantly, such optimized, ideal conditions (i.e., clear task description, no time pressure and therefore increased focus on task performance, optimized access to image presentation) are usually not available in a busy clinical routine, where clinicians need to quickly scroll through whole OCT volumes and often focus on central, vision‐related changes only.

A previous study evaluated the ability of retina specialists and a deep learning algorithm to correctly identify IRF/SRF in nAMD patients. In contrast to our study, however, the reference standard was not provided by the algorithm but by reading centre graders (Keenan et al., 2021). While continuous grader training, certification and supervision, as well as technical requirements, allow a relatively high degree of standardization at reading centres, eliminating all grading disagreements remains a challenge (Folgar et al., 2014; Michl et al., 2023). In the present study, the lowest specificity and thus highest rate of false‐positives were found for reading centre graders. This ‘overgrading’ might be due to their lower tolerance for retinal fluid (especially in single‐visit settings), which derives from their supervised work in clinical anti‐VEGF trials where high grading sensitivities are of the utmost importance. The greater tolerance of retinologists, on the other hand, might be attributed to their clinical experience in evaluating fluid dynamics between multiple visits in a given patient, as was the case in this study.

In contrast to fluid presence/absence, gauging fluid change at a volume level might be a task even retinologists are not accustomed to. This general unfamiliarity might also explain the similar performances of our professionals in distinguishing between fluid increase and decrease, as expressed by the AUCs. Examples showing IRF changes between −17 nL and +3 nL and SRF changes between −9.30 nL and +6.50 nL were associated with Sen > 0.80 and Spe > 0.87 among all graders. Interestingly, compared with the other groups, the retinologists were slightly less accurate at detecting decreases in IRF. While this could be merely due to chance, it might also be a consequence of the greater number of graders in this group and the resulting grading variability. This finding might further indicate that retinologists more generously describe conditions with little fluid change as stable.

While at first sight, our findings suggest a rather high accuracy of manually grading retinal fluid, they should be interpreted in a clinical context. In clinical practice, common treatment regimens such as T&E and PRN are largely based on the assessment of OCT disease activity and specify shortening of treatment intervals and reinjection once fluid reoccurs (Chakravarthy et al., 2020). Especially in a reactive rather than proactive treatment regimen such as PRN, missing even small amounts of IRF might delay retreatment and thus decrease the chance for visual and neurosensory recovery (Riedl et al., 2022). This problem is aggravated by substantial disagreements even between experienced ophthalmologists when assessing retinal fluid, especially in patients with little macular fluid or IRF only (Keenan et al., 2021; Toth et al., 2015). The unfavourable consequence might be fluctuations in retinal fluid volumes over the disease course that have been associated with worse visual outcomes and the development of irreversible macular fibrosis and geographic atrophy (Evans et al., 2020).

In advanced stages of nAMD, degenerative changes (i.e., degenerative cysts and outer retinal tubulations) in the macula make it difficult for eye care professionals to reliably identify small amounts of fluid and gauge their impact on vision (see Figure 4). Furthermore, a substantial number of patients may never reach a fluid‐free macula, even after intensive anti‐VEGF therapy (Core et al., 2022). This highlights the importance of an accurate identification and quantification of retinal fluid and its changes throughout life‐long monitoring. Measuring individual fluid levels as well as their impact on visual function is therefore key to defining the (re)occurrence of active, treatable disease and is an important step towards a truly personalized treatment regimen. This might be achieved by applying AI‐based fluid quantification, which allows objective and time‐efficient monitoring of fluid levels while eliminating grading inconsistencies between treating eye care professionals (Riedl et al., 2022; Schmidt‐Erfurth et al., 2020).

The application of any automated AI system to a diverse population is important for testing its applicability and benefit in the real world, which is why we applied the fluid monitor to OCT images of patients from a real‐world setting. Such a retrospective analysis, however, has potential drawbacks, such as missing data and selection bias. Furthermore, the relatively small sample size might limit the generalizability of our findings. As shown in Figure 2, there was a wide distribution of different fluid volumes for both IRF and SRF, indicating the inclusion of eyes at various treatment and/or disease stages (i.e., loading‐, maintenance‐, quiescent/degenerative phases). The small median for IRF (<5 nL = minimum volume to avoid exclusion) can be explained by eyes that showed <5 nL of IRF but were included due to their sufficiently large SRF volumes. On the upside, since these cases were still graded for both fluid types, this allowed us to study the (human) classification performance at all fluid levels.

The human grading in this study was compared with the output of the fluid monitor that served as the reference standard. The credibility and diagnostic accuracy of the latter have been demonstrated in numerous studies that have validated the fluid monitor and have led to its approval by the European Union Medical Device Regulation for clinical use in 2022. As an additional quality measure prior to the grading, all OCT images as well as the automated fluid segmentations were manually controlled by two ophthalmologists and grading supervisors from the VRC, who did not participate in the grading and confirmed their correctness without having to exclude any scans. Comparable kappa values between the professional groups and the fluid monitor further confirm its robustness.

In this study, AI‐based fluid assessment reached the accuracy of human experts from different professional backgrounds in eye care for grading retinal fluid and its change over time. Nevertheless, despite optimizing the methodology and time resources, manual human performance did not reach the high level of automated fluid monitoring. Importantly, the overall consistency between human and AI‐based disease activity assessments demonstrates the plausibility of automated monitoring. In the future, the efficient and successful management of rising patient numbers will depend on the adoption of such automated AI tools that are easily accessible to eye care professionals at all levels of patient care and offer substantial resource‐ and time savings (Schmidt‐Erfurth et al., 2022).

## AUTHOR CONTRIBUTIONS

MM: designed the study, collected data, analysed the data and wrote manuscript; BSG: designed the study, collected data, provided supervision of research and reviewed/edited manuscript; AG: analysed the data, reviewed/edited manuscript; FG: collected data, reviewed/edited manuscript; GM: collected data, reviewed/edited manuscript; OL: analysed the data, reviewed/edited manuscript; WB: collected data, reviewed/edited manuscript; SS: collected data, reviewed/edited manuscript; HB: designed the study, analysed the data, reviewed/edited manuscript; AS: analysed the data, reviewed/edited manuscript; USE: designed the study, provided supervision of research and reviewed/edited manuscript.

## CONFLICT OF INTEREST STATEMENT

BSG: Roche, Zeiss, Abbvie, Bayer (C); SS: Roche, Novartis, Bayer (C); USE: Apellis Pharmaceuticals, Bayer, AbbVie, Medscape, Allergan, Roche, Boehringer, Novartis, Galimedix, Aviceda Therapeutics, Annexon Bioscience, Topcon (C); Genentech, Kodiak, Novartis, RetInSight and Apellis Pharmaceuticals (F); All other authors declare no competing interests.

## ETHICS STATEMENT

The Ethics Committee of the Medical University of Vienna approved the VIBES registry and analysis performed in the present study (approval numbers: 2094/2018 & 2095/2018).

## Data Availability

Data are available on reasonable request.
